# Supplementary material for: Are carbon emissions trading and green financial instruments synergistic? -Comprehensive quantitative research based on content analysis
Source: PLoS One. 2024 Mar 7;19(3):e0298601. doi: 10.1371/journal.pone.0298601 (PMC10919601; doi:10.1371/journal.pone.0298601)
Supplement: S1 Data — (ZIP) [file pone.0298601.s001.zip › Data/地级市Z_F工作报告.zip!/─■╧─/╓╨╬└╩╨/└┤╫╘╣1⁄2╓┌║┼í╛┬φ┐╦╩2╛▌═°í┐_2005.docx]

各位代表：

现在，我代表市人民政府，向大会做政府工作报告，请予审议，并请市政协各位委员和其他列席人员提出意见。

一、2004年工作回顾

中卫市成立以来，全市人民在自治区党委、政府和市委的正确领导下，认真贯彻党的十六大和十六届三中、四中全会精神，牢固树立和落实科学发展观，坚持一手抓筹建、一手抓发展，圆满完成了市第一届人民代表大会第一次会议确定的各项目标任务。预计全市地区生产总值完成50.3亿元，增长12.6%。其中：第一产业12.3亿元，增长3.5%；第二产业23.4亿元，增长18%；第三产业14.6亿元，增长11.5%。地方财政收入完成3.29亿元，可比增长24.3%，其中市本级财政收入1.87亿元，可比增长26.7%。全社会固定资产投资完成55.5亿元，增长31.9%。城镇居民人均可支配收入6560元，增长10.3%。农民人均纯收入2320元，增长8%。城镇登记失业率为4.5%。人口自然增长率为12.9‰。

一年来，我们主要做了以下几个方面的工作：

（一）实施项目带动战略，工业经济发展势头良好

坚持把工业摆在经济工作的首位。以园区为载体，以项目为支撑，着力培育优势产业，发展壮大骨干企业。全市开工建设工业项目114个，完成投资11.7亿元。美利纸业林纸一体化项目由国家发改委批复实施；明巨电石、弘骞冶化、宁夏红集团、赛马集团中宁公司、天元公司等项目建成投产；科豪陶瓷、中鑫硅镁等新建、扩建项目正在加紧建设。全年规模以上企业完成工业总产值35.5亿元，占全市工业的75.2%；实现利税3.2亿元，占全市工业的 90%。配套建设了美利、宁新工业园区水、电、路等基础设施，美利工业园区南区主干道路和上水工程基本完成。城区东园330KVA变电所建成运营，中宁大战场330KVA变电所正在加紧建设。美利、宁新等工业园区共落实项目58个，总投资32.8亿元，完成投资近3亿元。出台《中卫市招商引资若干政策规定》等优惠政策，资本运作意识不断增强，服务环境进一步优化。全市共签订招商引资项目196个，协议投资60.2亿元，到位资金18.6亿元。全市工业完成增加值14.2亿元，同比增长16.8%，销售收入45.2亿元，同比增长20.4%。

（二）创新经营理念，城市建设步伐加快

完成中卫城市总体规划的修编报批工作，市区规划面积由30平方公里拓展到52平方公里，新区分区规划和控制性详细规划正在加紧修编。全市新建、续建城市建设项目131个，完成投资12.2亿元。市区中央大道、迎宾大道、平安大道等8条骨干道路正在建设；美利广场、供排水、供热、污水及垃圾处理等一批项目基本建成；新建、续建了向阳小区、阳光家园等14个商住小区，总建筑面积达116万平方米。中宁县人民广场、世纪花园、集中供热和海原县商业街、利民花园等城市建设项目进展顺利，都呈现出良好的发展态势。拓宽了融资渠道，与国家开发银行成功搭建了开发性金融合作平台。全市挂牌出让土地38宗，收缴和清欠土地出让金9387万元。充实城管队伍，加大城市综合执法力度，实施城市主要街道净化、亮化、美化工程，拆除影响市容市貌的违章和危旧建筑，城市面貌有了新变化。

（三）调整优化农业结构，农业和农村经济稳步发展

围绕增加农民收入，兑现粮食直接补贴等政策措施，加大财政金融支农力度，实施农业重点项目，农业结构战略性调整取得新进展。枸杞、草畜、设施蔬菜、鸡禽、马铃薯五大战略性主导产业和西甜瓜、生猪、优质米三大区域性特色产业规模和效益进一步提高，一批农产品加工、流通龙头企业辐射带动能力增强，优势农产品产业带基地初步形成。实施了城区镇罗5000亩无公害设施蔬菜和宣和百万只养鸡示范园区及枸杞、西甜瓜、草畜等基地项目建设。全市设施蔬菜达到4.2万亩，枸杞13万亩，西（甜）瓜16万亩，优质牧草118.5万亩。鸡、羊、猪、牛饲养量稳步提高，各类养殖园区达到185个。农业基础设施得到加强，引黄灌区续建配套与节水改造、山区小流域治理和治河治沟工程进展顺利。硬化乡村主干道路161条130公里，乡村交通环境明显改善。巩固封山禁牧成果，草畜产业稳步推进。

（四）大力发展旅游业，第三产业活力增强

完成了沙坡头边关湿地旅游区规划。建成了沙坡头“白马拉缰”保护工程等31个旅游项目。成功举办中国宁夏（沙坡头）大漠·黄河国际旅游节、法国人“丝绸之路”长跑等活动，旅游知名度进一步提高。沙坡头旅游区被国家体育总局授予“中国全民健身20大景观之一”称号，被中央电视台等新闻媒体评为中国10大最好玩的地方之一。全年旅游人数达到74.5万人次，直接收入达到2550万元。服务业等第三产业发展步伐加快。全市餐饮服务网点达到1.5万个，餐饮收入4.3亿元，饮食业更加兴旺和活跃。房地产发展势头强劲，交易额明显上升，城乡市场日趋活跃。财税工作超额完成任务。金融工作成效明显，全市金融机构存款余额达到61.6亿元，同比增长12.8%；贷款余额达到59.5亿元，同比增长12%。

（五）全面推进社会各项事业，人民生活水平不断提高

“科教兴市”战略深入实施。建立技术创新体系和运行机制，推动高新技术产业化，提高科技成果转化率，研发和创新能力进一步提升，企业核心竞争力不断增强。深入实施科技特派员创业行动。围绕优势特色农产品开发，建立了一批农业科技示范园区。科普工作取得新成绩。实施中卫可持续发展实验区建设工程。科技进步对经济发展的贡献率有了新提高。大力实施“两基”攻坚、世行贷款、教育信息化和现代远程教育等重点教育工程。加大优质教育资源整合力度，扩大城区和中宁高中办学规模，撤并一批学校和教学点，教育结构和学校布局进一步优化。不断深化素质教育，教育教学质量稳步提高。

文化卫生体育事业不断繁荣。推进文化体制改革，加强精品创作，规范文化市场秩序，文化事业全面发展。成功举办市庆活动，全年广场文艺演出120场次，群众文化丰富多彩。广播电视播出质量提高，收视频道增加，覆盖范围扩大。建成市医院门诊大楼、中医院住院部和疾控中心。建立市县乡三级疾病预防控制、突发公共卫生事件救治、重大疫情信息网络和卫生执法监督体系，公共卫生服务水平和应急能力进一步提高。新型农村合作医疗试点进展顺利。妇幼保健工作得到加强。开展全民健身运动，群众体育和竞技体育运动取得新成绩。重视做好老龄和残疾人工作。加强民兵预备役建设，支持中卫军分区、武警消防部队建设，认真做好军队转业干部、退役士兵安置和拥军优属工作，“双拥”共建活动深入开展。

人口资源环境工作成效显著。川区开展知情选择和优质服务活动，山区加快推进“少生快富”扶贫工程，人口和计划生育工作不断加强。严格保护土地资源，实现了耕地占补平衡。以水污染和大气污染治理为重点，工业污染治理取得明显成效，城市空气质量显著改善。统计、外事侨务、防震减灾、人防、气象等工作取得新成绩。

就业和再就业工作不断加强。全年新增就业岗位3970个，其中一次性购买“4050”人员再就业岗位400个，弱势群体的就业和再就业得到有效解决。千方百计加大社会保障资金投入，养老保险、失业保险、医疗保险统筹覆盖面进一步扩大。筹措资金近1300万元，解决了城区破产企业退养人员和下岗失业人员的医疗保险与养老保险问题。全年发放各类救济救灾资金4000万元。加大对农民工工资的清欠力度，维护了农民工的合法权益。实施沙坡头移民搬迁、贫困户养羊、万户菌草种植等扶贫开发项目，有组织地转移农村富余劳动力8万人次，农民外出务工收入2.5亿元。山区基础设施建设和特色产业开发投入加大。脱贫人口达到4.3万人，山区群众的生产生活得到改善。

（六）精神文明建设健康发展，民主法制建设不断加强

认真贯彻“三个代表”重要思想，牢固树立科学发展观和正确政绩观，深入学习十六届三中、四中全会精神，执政为民意识有效增强，行政能力不断提高。群众性精神文明创建活动蓬勃开展，社会主义思想道德建设继续加强，新人、新事、新风尚不断涌现，未成年人成长环境进一步优化。坚持社会主义物质文明、精神文明、政治文明协调发展，自觉接受人民代表大会及其常委会的监督，认真执行人大的各项决议决定。密切与人民政协的联系。广泛听取各民主党派、工商联、无党派人士和人民团体的意见建议。办理市人大代表议案2件、建议21条、政协提案176件。坚持职工代表大会制度，完善村民自治和城市居民自治，推行政务公开、村务公开和厂务公开，人民群众合法权益得到保障。加快推进政府法制建设，制定行政规范性文件5件。认真贯彻落实《行政许可法》和国务院《全面推进依法行政实施纲要》，清理公布行政许可事项197项，废止7项。深入开展“四五”普法教育，公民法律意识进一步增强。落实廉政建设责任制，加大从源头预防和惩治腐败力度，纠正部门和行业不正之风，加强行政监察和审计监督，政府系统廉政建设取得新成效。认真落实党的民族宗教政策和《民族区域自治法》，平等、团结、互助的社会主义民族关系得到巩固和发展。正确处理改革、发展、稳定关系，高度重视群众来信来访，强化社会治安综合治理，深入开展严打专项斗争，努力消除不安定因素，维护了社会稳定。

各位代表！

过去一年取得的成绩来之不易，这是全市上下、方方面面团结奋斗、辛勤劳动、无私奉献的结果。在此，我代表市人民政府，向全市工人、农民、知识分子、干部、解放军指战员、武警官兵、公安干警，表示崇高的敬意！向人大代表、政协委员、各民主党派、工商联、人民团体、离退休老同志，向关心和支持中卫发展的社会各界人士，表示衷心的感谢！

回顾过去一年，我们既要看到所做的工作和已取得的成绩，又要清醒地认识到前进道路上还有许多困难和问题。主要表现在：经济社会发展的体制性、机制性障碍依然存在；资金、电力、运力等生产要素供需矛盾比较突出；工业经济增长缺少大企业、大项目带动；一些城市建设项目进展缓慢；农民增收的渠道还不宽；城镇居民人均可支配收入仍然较低；一些部门工作作风还不够扎实等等。对这些困难和问题，我们将高度重视，认真解决。

二、2005年主要任务

各位代表，2005年是贯彻落实科学发展观、保持经济社会良好发展态势的重要一年，更是全面完成“十五”规划目标、衔接“十一五”发展的关键一年。今年政府工作的总体要求是：以邓小平理论和“三个代表”重要思想为指导，深入贯彻落实党的十六大及十六届三中、四中全会精神，以发展为主题，以建设生态旅游文化城市为目标，以项目为龙头，以创新为动力，坚定不移地推进工业化、城市化、农业产业化和旅游特色化，全面加快山川共济进程，千方百计提高人民生活水平，切实加强精神文明建设和民主法制建设，促进全市经济和社会各项事业全面协调可持续发展。主要预期目标是：全市地区生产总值达到58亿元，增长12%。其中：第一产业13亿元，增长4 %；第二产业28亿元，增长18%；第三产业17亿元，增长10%。地方财政收入达到3.07亿元，可比增长13.6%，其中市本级财政收入1.67亿元，可比增长13.6%。全社会固定资产投资达到68亿元，增长22.5%。城镇居民人均可支配收入达到7200元，增长9.5%。农民人均纯收入达到2440元，增长5.3%。城镇登记失业率控制在4.5%以内。人口自然增长率控制在13‰以内。

重点抓好八个方面的工作：

（一）全力推进新型工业化，提升经济发展总量

实施工业强市战略，提高工业发展水平，是我市实现跨越式发展的重大战略选择。今后一个时期，全市工业发展的总体要求是：突出项目支撑，突出园区建设，突出招商引资，切实提高工业增长的质量和效益，走出一条科技含量高、经济效益好、资源消耗低、环境污染少、人力资源优势得到充分发挥的新型工业化路子。战略重点是：全力实施“三个百万吨工程”，即实施百万吨造纸、百万吨电石、百万吨以电石为原料的PVC及下游产品工程。通过3—5年，实现年造纸135万吨，电石和以电石为原料的PVC及下游产品各100万吨，年产值达到200亿元，届时中卫将成为宁夏乃至西部重要的能源重化工基地。年内，力争造纸、电石、以电石为原料的PVC及下游产品生产能力分别达到36万吨、50万吨、30万吨。

培育壮大优势骨干企业。围绕造纸、酿酒、冶金化工、建筑建材、农副产品加工五大优势产业，培育壮大美利纸业、秦毅集团、宁夏红集团、明巨电石、圣得华镁业、明盛染化、赛马集团中宁公司、赢海集团、赢嘉实业、大河机床等一批技术创新能力强、管理水平高、具有持续盈利能力和抗风险能力的优势骨干企业群体。年内，力争建成1家产值超过20亿元、2家产值超过10亿元、5家产值超过5亿元、10家产值超过亿元的大企业、大集团。

加快园区基础设施和入园项目建设。全面提高美利、宁新工业园区水、电、路、通信、绿化等基础设施标准。建成美利工业园区南区供水工程。完成中宁大战场330KVA变电所和美利工业园区2个110KVA变电所建设。引导民营企业、外资企业和新兴产业向园区集中。加快项目入园进度，重点抓好美利纸业热电厂、30万吨涂布白卡纸、10万吨杨木化机浆，宁夏日盛电化公司8万吨电石、6万吨烧碱、5万吨PVC和宁夏宝塔集团45万吨电石等145个工业项目。

大力发展非公有制经济。毫不动摇地鼓励、支持和引导非公有制经济发展。要不断加大扶持力度，从政策、资金和人才上鼓励引导非公有制企业加快转换，创新管理方式，加快技术改造，提升产品技术含量和档次，形成新的竞争优势，力争非公有制经济增加值占地区生产总值、上缴税金占地方财政收入、吸纳劳动力占城镇就业三个比重均有明显上升。

全方位推进招商引资。实行更加优惠的政策，依托资源优势，有针对性地优选一批招商项目，主动出击，与区内外大企业、大集团寻求合作，扩大利用外资规模，提高利用外资水平。进一步加强与沿海发达地区的经济技术合作，努力实现互利双赢、共同发展。抓好已签约项目的跟踪落实，提供全程服务，力争更多的项目落户中卫。创新招商方式，大力推行企业招商、以商招商、中介招商、网络招商和资源招商，促进招商工作市场化、企业化、社会化。全年引进各类招商项目超过100个，协议资金超过56亿元。

（二）大力推进城市化，不断提升城市品位和档次

开展“城市建设年活动”。按照“拉大城市框架、突出新区建设、加快旧城改造、配套基础设施”的要求，强化产业支撑，完善城市功能，丰富文化内涵，营造和谐环境，多方吸引投资，全力加快城市化进程。

实施新区建设战略。完成新区规划、重点地段控制性详细规划的编制工作，严格规划审批，做到“规划一张图、审批一支笔、建设一盘棋”，确保城市规划建设的严肃性。将市区建设主战场转移到新区，将二、三类工业逐步从市区迁出。重点抓好中央大道延伸、滨河大道延伸、迎宾大道、平安大道、应理南街延伸、卫谢南路延伸、绕城北环路和彩虹大道8条道路和新区生态湖、水系、沿河景观带建设。规划建设新墩路、东环路、西环路。改造整治鼓楼东西街、应理南北街、香山街、文昌街、长城街、中山街和商业南北街人行道。配套市区供热、供排水、供电及教育、文化、卫生等基础设施建设。建成中卫一中、市医院传染病区和急救中心。迁建中卫市场和市第二人民医院，开工建设“五馆三中心”（文化馆、图书馆、博物馆、展览馆、科技馆和青少年德育教育中心、老干部活动中心、职工活动中心）。积极筹建中卫高等职业技术学院。加快建设行政中心、新闻中心、电力调度中心、公、检、法、农牧林业、工商、国税、地税等27个公共项目，逐步将老城区部分公共设施迁移到新区；新建、续建世纪花园、广厦新村、丽景园等16个商住小区，形成新的生态型办公区和商住区。科学合理地编制土地利用总体规划，调整城区基准地价。强化城市环境综合整治，优化人居环境。加强市政公用事业管理，切实提高城市管理水平。

加快城镇建设。中宁、海原县城建设要科学规划，体现特色，稳步推进，不断提高发展档次和水平。因地制宜，重点发展一批工矿型、交通枢纽型、商贸型、旅游型等中心小城镇。充分发挥城市对农村的辐射带动作用，努力打破城乡分割的二元结构，推动产业和人口向城镇聚集，力争年内城市化率提高五个百分点以上。努力改善农村住房条件，加强村庄环境综合整治。抓好石营公路沿线两侧环境整治，建设现代化新农村。

（三）积极推进农业产业化，促进农业和农村经济全面发展

农业是安天下、稳民心的战略产业。要认真贯彻中央一号文件精神，坚持城乡统筹，以川济山、山川共济；坚持“多予少取放活”的方针，进一步加大对农村的投入力度；以增加农民收入为核心，以提高农业综合生产能力为主题，努力实现农业由计划型、数量型向质量型、效益型和市场型转变。

大力培育优势特色产业。认真实施《优势特色农业发展五年规划》，加快五大战略性主导产业和三大区域性特色产业向优势产区集中。在稳定粮食生产的基础上，新发展枸杞3万亩，累计达到16万亩，建设卫宁灌区枸杞观光长廊，打造“世界枸杞之都”。加快无公害设施蔬菜基地建设，新建千亩集中连片的示范基地5个，新增设施蔬菜1.2万亩，把我市建成宁夏的“寿光”乃至西部的“寿光”。加快城区永康百万只鸡生态养殖园区建设，鸡饲养量新增300万只，累计达到1500万只，把我市建成西部最大的鸡禽养殖基地。羊、猪、牛饲养量分别新增25万只、25万头、2万头，累计分别达到200万只、130万头、20万头。同时，狠抓山区特色种植，马铃薯种植面积达到40万亩；新增优质牧草21.5万亩，累计达到140万亩；新增压砂西（甜）瓜10万亩，累计达到26万亩，把我市建成西部最大的绿色西（甜）瓜基地。

进一步提高农业产业化水平。发展农副产品深加工，培育壮大宁夏红集团、赢嘉淀粉、新宁乳业、金泉草业等一批带动能力强的龙头企业。高度重视农副产品流通，扶持一批农副产品流通协会和经纪人队伍，在西部大中城市设立直销窗口，解决农民“卖难”问题。大力推进农业标准化生产，发展无公害食品、绿色食品和有机食品生产基地，完善农业标准体系和农产品质量监测、动物疫病防治体系建设，提高农产品质量。

加强农业基础设施建设。开展农田水利基本建设，继续实施引黄灌区续建配套、山区小流域治理和治沟治河工程，提高农业抵御自然灾害的能力。开工建设红三公路二期工程，硬化城区乡村道路150公里以上，3年内超过500公里，不断改善农村交通环境。

加大扶贫开发力度。继续加大资金和政策扶持，稳步扩大扶贫成果。以整村推进为切入点，认真实施千村扶贫开发工程和十万贫困户养羊工程，继续抓好部门定点帮扶工作，努力改善山区群众生产生活条件，增强自我发展能力。以实施劳动力培训工程为重点，大力开展劳务输出，加快农村富余劳动力转移，增加农民非农产业收入，全年完成劳务输出9万人次。稳步推进退耕还林还草工程，培育壮大后续支柱产业。巩固禁牧封育成果，恢复生态植被。

（四）突出抓好旅游业，加快发展第三产业

大力发展立体旅游和网络旅游。围绕游、行、吃、住、购、娱六要素，依托优势，打造精品，加强营销，规范服务。挖掘旅游资源，加快景区建设，努力展现中卫塞上江南、黄河文化、回乡风情和大漠风光的神奇魅力。继续办好“中国宁夏（沙坡头）大漠·黄河国际旅游节”等节事活动，提高旅游知名度。全年接待游客85万人次，旅游直接收入3230万元，间接收入1.84亿元。

繁荣发展现代服务业。要高度重视，制定规划，完善政策，提升整体水平，使服务业成为我市新的经济增长点。着力培育现代物流、信息咨询、中介服务、电信邮政、金融保险等新兴产业，改造提高商贸、餐饮、酒店、仓储、交通运输等传统服务业，加快发展社区服务业。学习借鉴现代理念和管理方式，建设大型现代化连锁超市。积极促进住房、汽车、健身等消费，提升消费档次。

（五）深化各项改革，切实解决经济运行中的矛盾和问题

进一步落实农村各项改革政策。落实好国家和自治区对种粮农民的直接补贴、取消农业税等政策。加强粮食市场监管，深化粮食流通体制改革。全面推行灌区供水体制和水价制度改革。健全农民负担监督管理机制，加强村级财务管理。着力化解镇村债务，切实减轻农民负担。

推进企业改革。围绕建立现代企业制度和完善的公司法人治理结构，坚定不移地深化以产权制度改革为主要内容的企业改革，加快改制企业职工身份置换。进一步深化企业内部用工、人事和分配制度改革。规范企业经营行为，加强企业资金、技术、人才、营销管理，促其做大做强。

加快事业单位改革。改革事业单位用人和分配制度，全面推行聘任制，建立多元化分配制度。实施事业单位分类管理和改革，对经营开发服务类事业单位，实行改企转制。机关所属经营类单位，率先脱钩改制。

深化国有资产管理和投资体制改革。建立和完善公平公正的经营业绩考核体系、科学严密的统计评价体系和行之有效的激励约束机制，依法加强国有资产监管，严防国有资产流失，确保国有资产保值增值。全面推行项目核准制和备案制，落实企业投资自主权。建立投资决策责任制，规范政府投资行为。

落实财税金融改革政策。认真落实积极的财政政策和货币政策。严格部门预算约束，扩大国库集中支付范围，加大会计集中核算工作力度，提高财政资金使用效益。推行依法治税，加强税收征管，加大稽查力度，严厉打击偷、逃、骗、抗税行为。加强银政、银企合作，支持金融企业扩大信贷规模，拓展服务领域，促进地方经济发展。

（六）加快社会事业进步，促进经济社会协调发展

加快科技和教育创新。建立和完善以企业为主体的技术创新体系。发挥科技园区的示范作用，努力提高区域科技创新能力。认真贯彻《国家科技基础条件建设纲要》，增加科技投入，改善科研基础条件。继续实施中卫可持续发展实验区建设工程。深入开展科技特派员创业行动，完善科技创新体系，促进科技成果转化。做好知识产权保护、专利和科普工作，推进各类实用技术的普及和应用。坚持把教育放在优先发展的地位。巩固提高城区和中宁县“普九”成果，加快海原县“普九”攻坚步伐。积极发展职业教育和民办教育，培养更多实用型人才。鼓励和支持社会力量办学。优化配置教育资源，全面加强素质教育，进一步完善信息技术教育网络。大力普及高中阶段教育，做好高中新课程改革工作，不断提高教育教学质量。

发展文化卫生体育等事业。实施精品战略，繁荣文艺创作，大力开展群众喜闻乐见的文化娱乐活动。加强公共文化娱乐场所监管，营造健康向上的文化环境。推进文物保护、新闻出版和广播电视事业。建立和完善公共卫生信息网络、疾病预防控制、医疗救治和卫生监督执法体系，增强突发性公共卫生事件的应急处置能力。继续做好新型农村合作医疗试点工作，有效改善农村医疗卫生条件，提高全民卫生健康水平。开展全民健身活动，提高竞技体育水平。加紧备战2006年全区运动会。维护妇女儿童合法权益。关心支持老龄、残疾人事业。继续做好外事侨务、防震减灾、气象等工作，推进社会事业全面发展。

促进人口与资源环境协调发展。严格落实人口与计划生育目标责任制，继续推进“少生快富”扶贫工程，切实降低人口出生率，坚决控制人口数量，提高人口质量。强化生态环境监管，严格控制主要污染物排放。新的招商引资项目和迎水地区企业扩建项目，原则上都要向工业园区集中。依法保护和合理利用国土资源。加强矿产资源开发的规划和管理，坚决制止滥采乱挖和严重浪费现象。加强水资源保护和节约利用。

（七）加大就业和社会保障力度，努力提高人民生活水平

切实做好就业和再就业工作。把促进扩大就业放在经济社会发展更加突出的位置。认真落实有关政策，促进下岗失业人员自谋职业、自主创业。健全完善再就业援助制度，抓好劳动力市场建设和管理工作，加快建立公共就业服务体系。继续做好大中专毕业生就业、复转军人安置、城镇新增和农村富余劳动力就业工作。

进一步完善社会保障体系。认真做好“两个确保”和“三条保障线”的衔接，继续完善养老、失业、医疗保险制度，启动工伤保险，完善社会保障体系，努力做到应保尽保。加大社保基金的征缴、扩面和清欠力度，增加基金总量。积极探索建立农村最低生活保障制度。进一步搞好新型农村合作医疗制度试点。逐步建立自然灾害救助、农村社会救助、城市社会救助制度，构筑功能完善、保障有力的城乡社会救助体系。

维护好群众的合法权益。完善征地程序和安置办法，严格城市房屋拆迁管理，做好安置补偿工作。强化劳动执法监察，搞好劳动安全保护，严格执行最低工资标准。切实解决好建筑领域拖欠工程款和农民工工资问题。规范和整顿市场经济秩序，严厉打击制售假冒伪劣商品的违法行为。探索建立食品药品安全监管长效机制，继续推进放心食品药品工程。高度重视安全生产工作，认真落实安全生产责任制。加强对矿山、道路交通、公共场所的安全监管，严格管理易燃、易爆、危险化学品，杜绝和避免重特大事故的发生。认真执行安全事故责任追究制，对玩忽职守、漠视人民生命财产安全，造成重大安全事故的责任者，要从严查处。

（八）加强精神文明和民主法制建设，促进“三个文明”全面发展

认真开展以实践“三个代表”重要思想为主要内容的保持共产党员先进性教育活动。弘扬以爱国主义为核心的民族精神和以改革创新为核心的时代精神。加强社会公德、职业道德和家庭美德教育，在全社会倡导爱国守法、明礼诚信、团结友善、勤俭自强、敬业奉献的基本道德规范。切实抓好青少年的思想品德和心理素质教育，营造有利于青少年健康成长的思想文化环境。深入开展群众性精神文明创建活动。加强科普宣传，反对封建迷信，倡导和树立科学、文明、健康的生活方式，提高全社会文明程度。

坚持党的领导、人民当家作主和依法治国的有机统一。认真执行人民代表大会及其常委会的决定和决议，自觉接受监督。支持人民政协履行好政治协商、民主监督、参政议政职能。认真办理人大代表议案、建议和政协提案。主动听取各民主党派、工商联和社会各界的意见和建议。加强基层民主政治建设，推进村务公开、厂务公开，保障基层群众依法行使选举权、知情权、参与权、监督权等民主权力。深入贯彻党的民族政策，巩固和发展平等团结互助的社会主义民族关系。全面贯彻党的宗教信仰自由政策，依法管理宗教事务，坚持独立自主自办的原则，积极引导宗教与社会主义社会相适应。

认真开展“平安中卫”创建活动。全面落实社会治安综合治理的各项措施，严厉打击各种刑事犯罪活动和经济犯罪活动，扫除社会丑恶现象，保障人民群众安居乐业。做好“四五”普法的验收和“五五”普法的衔接工作。抓好以宪法为核心的法制宣传教育，增强全民的法律意识和全社会的法制观念。加强律师、公证等法律服务，做好法律援助和人民调解工作。创新信访工作机制，积极做好预防和妥善处置群体性事件工作。加强国防教育，搞好民兵预备役建设。深入开展“双拥”共建，认真落实拥军优属政策，巩固发展军政军民团结，努力创建全区、全国“双拥模范城”。

三、加强政府自身建设

各位代表，履行好政府职责，肩负起人民的重托，加快实现我市经济社会跨越式发展，必须牢固树立和落实科学发展观，按照党的十六届四中全会的要求，不断加强政府自身建设，努力提高行政能力，适应新形势，增长新本领，树立新形象，创造新业绩。

与时俱进，建设学习政府。全体公务员特别是各级领导干部要顺应时代前进的潮流，深入学习党的十六大和十六届三中、四中全会精神，把学习贯彻“三个代表”重要思想不断引向深入。学习政治、经济、文化、科技、法律等方面的最新知识和理论，提高认识世界、解决问题、做好实际工作的能力。坚持理论联系实际、学以致用的良好学风，强化创新意识，不断推进制度创新、科技创新、文化创新以及其他各方面的创新，努力使政府工作体现时代性、把握规律性、富于创造性。进一步抓好各类专业培训，开展公务员行为规范教育，完善公务员考核、录用和奖惩制度，推行竞争上岗和岗位轮换，不断提高公务员队伍的综合素质。

以人为本，建设服务政府。坚持把最广大人民的根本利益作为制定政策、开展工作的出发点和落脚点，正确反映和兼顾不同方面群众的利益。高度重视和维护人民群众最现实、最关心、最直接的利益，坚决纠正各种损害群众利益的行为。始终保持良好的状态和全新的观念，始终保持不怕苦、敢吃苦的精神，深入到困难群众中去，深入到矛盾多的基层单位中去，真心实意为群众排忧解难，尤其对弱势群体倾注更多的关爱，让千家万户有更多的笑容，有更多的温馨。

求真务实，建设高效政府。政府各部门特别是各级领导干部必须肩负起时代赋予的重任，切实增强全局观念，识大体、顾大局，做到令行禁止、政令畅通。要大兴调查研究之风，精文简会，减少领导干部参加庆典和迎来送往，不搞形式主义的达标评比活动。要进一步搞好作风建设，加快环境创新，促进工作落实，充分发扬激情创业、热情干事的精神，努力克服思想观念、工作思路、工作节奏与市委、政府的不适应，大力倡导争创一流的志气、开拓创新的才气、敢说敢干的硬气和襟怀坦荡的正气，促进政府工作快捷高效。

依法行政，建设法治政府。认真贯彻《行政许可法》和国务院《全面推进依法行政实施纲要》。按照合法行政、合理行政、高效便民、诚实守信、权责一致的要求，严格依照宪法和法律规定的权限和程序行使权力、履行职责。进一步加强政府法制建设，提高依法行政的能力和水平。建立健全科学民主决策机制，坚持重大事项公众参与、专家论证和政府决策相结合的行政决策机制。建立健全行政执法责任制，规范执法程序，以硬手段治理软环境。

廉洁奉公，建设清廉政府。从完善机制和制度入手，进一步规范事权、财权和人事权。加强政府系统内部监督，支持监察、财政、审计部门依法履行监督职责，对招投标、重大项目变更、专项资金管理等一切经济活动进行全程跟踪监督，从源头上预防和治理腐败。开展行风评议，纠正行业不正之风。充分发挥法律监督、民主监督、群众监督和舆论监督作用，提高管理水平，降低行政成本，力戒奢侈浪费，以实际行动取信于民。

各位代表！中卫作为宁夏最年轻的地级市，前景光明，任重道远，加快发展，时不我待。让我们更加紧密地团结在以胡锦涛同志为总书记的党中央周围，高举邓小平理论和“三个代表”重要思想伟大旗帜，在自治区党委、政府和市委的领导下，抢抓机遇，开拓进取，扎实工作，乘势而上，为实现中卫经济社会全面协调可持续发展而努力奋斗！
